# A Novel Benzothiazole Derivative YLT322 Induces Apoptosis via the Mitochondrial Apoptosis Pathway *In Vitro* with Anti-Tumor Activity in Solid Malignancies

**DOI:** 10.1371/journal.pone.0063900

**Published:** 2013-05-30

**Authors:** Song Xuejiao, Xia Yong, Wang Ningyu, Zhang Lidan, Shi Xuanhong, Xu Youzhi, Ye Tinghong, Shi Yaojie, Zhu Yongxia, Yu Luoting

**Affiliations:** 1 State Key Laboratory of Biotherapy and Cancer Center, West China Hospital, West China Medical School, Sichuan University, Chengdu, China; 2 Department of Pharmaceutical and Bioengineering, School of Chemical Engineering, Sichuan University, Chengdu, China; The Ohio State University, United States of America

## Abstract

Benzothiazole derivatives are known for various biological activities, and their potency in cancer therapy has received considerable attention in recent years. YLT322, a novel synthesized benzothiazole derivative, exhibits potent anti-tumor activity *via* inducing apoptosis both *in vitro* and *in vivo*. In this study, we found that YLT322 showed growth inhibition against a broad spectrum of human cancer cells and induced apoptosis of HepG2 cells in a dose- and time-dependent manner. The occurrence of its apoptosis was associated with activation of caspases-3 and -9, but not caspase-8. YLT322 increased the expression of Bax, decreased the expression of Bcl-2, and induced the release of cytochrome *c* which activates the mitochondrial apoptotic pathway. The down-regulation of phosphorylated p42/44 MAPK and phosphorylated Akt was also observed. Moreover, YLT322 suppressed the growth of established tumors in xenograft models in mice without obvious side effects. Histological and immunohistochemical analyses revealed an increase in TUNEL and caspase-3-positive cells and a decrease in Ki67-positive cells upon YLT322. These results suggest that YLT322 may be a potential candidate for cancer therapy.

## Introduction

Hepatocellular carcinoma (HCC) is the fifth most common cancer worldwide and the third cause of cancer-related death [1], [2]. Chemotherapy is the most usual treatment approach, in addition to resection and liver transplantation; however, it does produce satisfactory results because of the resulting poor response rates, severe toxicities and high recurrence rates [3], [4]. Resistance to apoptosis might account primarily for the resistance of tumor cells to chemotherapy and for cancer progression [3], [5]. Modulation of apoptosis sensitivity of cancer cells has emerged to be a promising strategy to induce cell death in cancer cells and indeed, most chemotherapeutic drugs could tumors by triggering cancer cell apoptosis [6].

Generally, drugs induce apoptosis in cancer cells through two pathways: cell death receptor-mediated extrinsic pathway and mitochondrial-mediated intrinsic pathway. In the extrinsic pathway, the ligation of so-called death receptors results in the activation of the protease caspase-8 which then cleaves and activates downstream effectors caspase-3 and/or -7, resulting in chromatin condensation, DNA degradation, cell shrinkage and formation of apoptotic bodies [7]. In the intrinsic pathway, Bcl-2 family members are the key regulators of apoptosis. Once the anti-apoptotic members of this family are inhibited and/or the pro-apoptotic members are activated, mitochondrial integrity is disrupted and cytochrome *c* is released. As a consequence of these changes, cytochrome *c* interacts with the Apaf-1 (apoptotic protease activating factor - 1) and ATP, and then binds to pro-caspase-9. This interaction results in the cleavage of pro-caspase-9, which in turn activates the effector caspase-3 and/or -7 [8]. Various anti-cancer agents have been shown to induce apoptosis through the intrinsic pathway [9].

Because of their wide range of biological activities, a number of benzothiazole derivatives have attracted interest for their potential pharmacological applications [10]–[12]. In recent years, extensive study has focused on assaying novel benzothiazole derivatives for anti-tumor activities. Our research group has been interested in the design, synthesis, screening and biological evaluation of novel benzothiazole derivatives as potential anticancer agents. Among these, 2-Chloro-N-(2-(2-(5-methylpyridin-2-ylamino)-2-oxoethylthio)benzo[d]thiazol-6-yl) acetamide (YLT322) displays robust anti-proliferative activity *in vitro* [13]. In this study, we demonstrated that YLT322 can induce apoptosis in human hepatocellular carcinoma cells *via* the mitochondrial apoptotic pathway and the down-regulation of phosphorylated Akt/MAPK, and also inhibit tumor growth *in vivo* by inducing apoptosis.

## Materials and Methods

### Drugs and reagents

**Figure 1:**
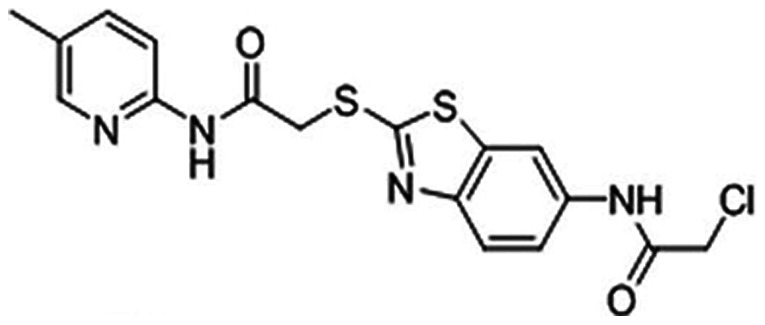
The chemical structure of YLT322.

2-Chloro-N-(2-(2-(5-methylpyridin-2-ylamino)-2-oxoethylthio)benzo[d]thiazol-6-yl) acetamide (YLT322) (Fig. 1) was synthesized previously by our group (State Key Laboratory of Biotherapy, Sichuan University, Sichuan, China) [13] and the structure was confirmed by 1H-NMR, 13C-NMR and HRMS (ESI). Purity (98%) was measured by HPLC analysis. YLT322 was dissolved in dimethyl sulfoxide (DMSO) at a stock concentration of 10 mM and stored at -20°C. For all *in vitro* assays, the working dosage was freshly diluted in relevant medium with a final DMSO concentration of less than 0.1%.

3-(4,5)-dimethylthiahiazo(-z-y1)-2,5-di-phenytetrazolium bromide (MTT), Rhodamine-123 (Rh123), Hoechst 33342, dimethyl sulfoxide (DMSO) and propidium iodide (PI) were purchased from Sigma Chemical Co. (St. Louis, MO). Annexin V-FITC apoptosis detection kit was purchased from Roche (Indianapolis, IN). The antibodies against caspase-3, caspase-9, Bax, Bcl-2, cytochrome c, Akt/p-Akt, p44/42 MAPK (mitogen-activated protein kinase)/p-p44/42 MAPK, and COX-4 were purchased from Cell Signaling Technology Company (Beverly, MA). Antibody against caspase-8 was from Thermo Scientific (Breman, Germany) and antibody against β-actin was obtained from Santa Cruz Biotechnology Company (Santa Cruz, CA). TUNEL (the terminal deoxynucleotidyl transferase mediated dUTP nick end labeling) assay kit was purchased from Promega Company (Madison, WI). Z-VAD-FMK (Caspase Inhibitor), LY294002 (PI3K/AKT Inhibitor), PD98059 (MAPK Inhibitor) were purchased from Beyotime (Beijing, China) and Ac-LEHD-FMK and Ac-IETD-FMK were from Keygen (Nanjing, China).

### Cell culture

The human HCC SMMC-7721,Bel-7402 and Bel-7404 cell lines were obtained from the China Center for Type Culture Collection (CTCCC,Wuhan, China). All other cell lines were acquired from the American Type Culture Collection. Cells were propagated in DMEM or RPMI 1640 medium (Gibco BRL, Grand Island, N.Y.) supplemented with 10% fetal bovine serum (FBS; Gibco, Auckland, N.Z.), 100 units/mL penicillin and 100 units/mL streptomycin under humidified conditions with 5% CO_2_ at 37°C. No further authentication was conducted for tumor cell lines. Cells without YLT322 treatment served as a vehicle group.

### Cell proliferation assay

Cell viability after YLT322 treatment was performed by the MTT assay. Briefly, the exponentially growing cells (3–5×10^3^ cells/well) were seeded in 96-well plates and cultured for 24 hours. After treatment with various concentrations of YLT322, a volume of 20 µl of MTT solution (5 mg/ml) was added to each well and incubated for another 2–4 hours at 37°C. Then the medium was discarded and the formazan salt was dissolved with 150 µl DMSO for 15–20 minutes. The absorbance of each well was measured with Spectra MAX M5 microplate spectrophotometer (Molecular Devices) at 570 nm wavelength, and the median inhibitory concentration (IC_50_) of each cell line was calculated. Three replicate wells were used for each analysis. The results were obtained from three separate experiments.

### Colony formation assay

To test the survival of HepG2 treated with YLT322, the cells were plated (6–10×10^4^ per well) in a six-well plate and incubated overnight at 37°C. After 48 h exposure to various concentrations of YLT322, the cells were cultured for another 12 days with fresh medium and subjected to a clonogenic assay as previously described [14].

### Morphological analysis after Hoechst staining

Morphological changes associated with apoptosis in HepG2 cells were detected by Hoechst 33342 staining. Briefly, the cells were plated onto 18-mm cover glass in 6-well plates for 24 h and then treated with YLT322 for another 24 h. After treatment, cells were rinsed with cold PBS and fixed in paraformaldehyde solution for 20 minutes. The cells were stained with Hoechst 33342 solution (5 u g/ml) followed by PBS washing and examination under fluorescence microscope (Zeiss, Axiovert 200, Germany) to identify the nuclear morphology of apoptotic cells.

### Apoptosis analysis by flow cytometry (FCM)

To further confirm the apoptosis inducing effect of YLT322, Annexin V-FITC apoptosis detection kit was used. Briefly, after treatment with different concentrations of YLT322 for 12, 24 and 48 h as described above, cells were harvested and washed with cold PBS twice. After centrifugation, cells were stained with Annexin V-FITC and PI, and then analyzed with FCM (Becton–Dickinson, USA).

Furthermore, to confirm whether caspases, AKT and p44/42 MAPK are involved in YLT322-induced apoptosis in HepG2 cells and to determine which pathways are important, we treated cells with or without 2 µM YLT322 combined with 20 µM Z-VAD-FMK (cell-permeable caspase inhibitor), 50 µM Ac-LEHD-FMK (caspase-9 inhibitor), 50 µM Ac-IETD-FMK (caspase-8 inhibitor), 50 µM LY294002 (PI3K/AKT Inhibitor) or 50 µM PD98059 (MAPK Inhibitor). Annexin V/PI staining was analyzed by FCM. We also studied the apoptosis inducing effect of YLT322 in other hepatocellular carcinoma cells, including Bel-7402, Bel-7404 and SMMC-7721 by PI staining.

### Mitochondrial membrane potential (ΔΨ) assay

Changes in mitochondrial transmembrane potential (ΔΨ) were evaluated by staining cells with Rh123 as described previously [15]. Cell culture and drug treatment were done as described above. The harvested HepG2 cells were washed with cold PBS after incubation with Rh123 (5 µg/ml) at 37°C for 30 min in the dark and then measured by flow cytometry .

### Western blot analysis

Cells were lysed in RIPA buffer after treatment with YLT322 for 48 h and the lysates were centrifuged at 13,000 g for 15 min at 4°C. The supernatant was harvested and the protein concentration was measured by the Lowry method. Equal amounts of total proteins were subjected to SDS-PAGE and transferred onto polyvinylidene fluoride membranes. After electrophoresis, the membranes were blocked for 1.5 h at room temperature and incubated overnight at 4°C with the respective primary antibodies followed by the secondary antibody conjugated to horseradish peroxidase. The immunostaining signal was detected by the enhanced chemiluminescence system (Amersham, Piscataway, NJ).

### Subcutaneous xenograft models

The study procedures were approved and conducted in accordance with the Animal Care and Use Committee of Sichuan University. 100 µL tumor cell suspension containing between 5×10^6^ and 1×10^7^ cells were injected subcutaneously into the right flank of the seven-week-old female BALB/c athymic nude mice. When tumors reached an average volume of 100 mm^3^, the mice were randomly divided into groups of 5 to 6. Animals were given YLT322 (37.5–150 mg/kg) or vehicle once daily by intraperitoneal injection. Tumor size and body weight were measured every three days, and clinical symptoms were observed daily. The tumor volume was calculated according to the following formula:V = (length×width^2^/2).

### Immunohistochemistry and TUNEL analysis

After treatment for seven days, tumors of HepG2 models were removed, fixed, routinely processed and embedded in paraffin. The sections containing tumors were stained with H&E or treated with specific antibodies for immunohistochemistry analysis. Apoptotic cells in the tumor tissue were identified by terminal deoxynucleotidyl transferase-mediated dUTP nick end-labeling (TUNEL) staining using an apoptotic cell detection kit.

### Sub-acute toxicity test

A sub-acute toxicity test was performed in BALB/C mice by oral administration with 2 g/kg YLT322. The clinical symptoms of the animals, including mortality, body weight and movement, were observed once a day for 14 days. Blood was obtained for hematological and serum biochemistry analysis by Hitachi 7200 Blood Chemistry Analyzer and a Nihon Kohden MEK-5216K Automatic Hematology Analyzer.

### Statistical analysis

Data are expressed as means ± SD or SE from three independent experiments. Student's test was used to assess the statistical significance of difference between groups. In all statistical analysis, a statistically significant difference was defined as a P value of <0.05.

## Results

### YLT322 inhibited proliferation of cancer cell lines

**Figure 2:**
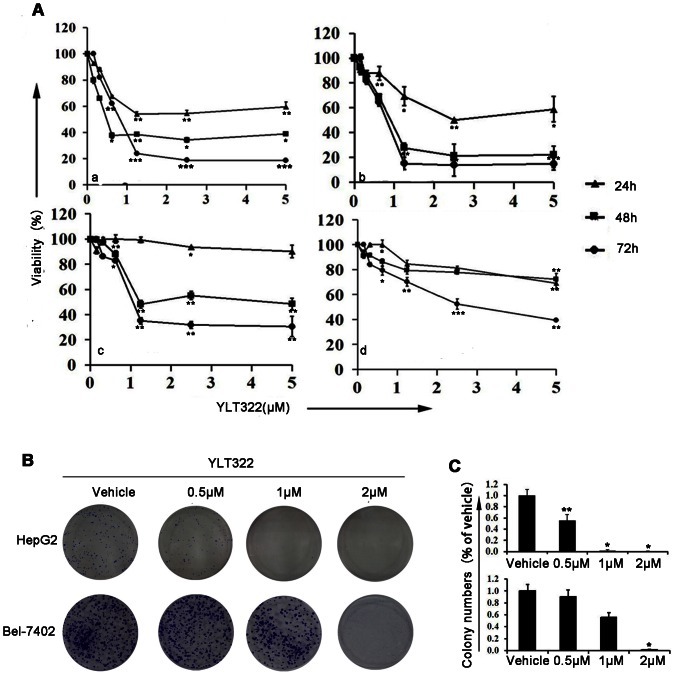
Inhibition of cell growth and colony formation in human cancer cell lines by YLT322 *in vitro*. **A.** Cells (a:HepG2; b:Bel-7402; c: Bel-7404;d:SMMC-7721) were treated with concentrations of YLT322 ranging from 0.15 to 5.0 µM, for 12 to 48 hours and cell viability was determined by MTT assay . Data are expressed as mean ± SD. for at least 3 independent experiments. (*p<0.05; **p<0.01; ***p<0.001) **B.** Effects of varying concentrations of YLT322 on colony formation of HepG2 and Bel-7402 cells after two weeks treatment. **C.** Statistical results of colony-forming assays presented as surviving colonies (percentage of untreated control). Data are expressed as mean ± SD. for at least 3 independent experiments. (*p<0.05; **p<0.01; ***p<0.001).

In order to determine whether YLT322 possesses the potential to be an effective anti-cancer agent, we first examined whether YLT322 exerts a growth inhibition effect on cancer cells by treating a panel of 24 established cancer cell lines of different histotypes with YLT322 for 48 hours and then assaying cell viability by MTT assay. YLT322 exhibited a significant cell viability inhibition with an IC_50_ between 0.39 µM and 7.70 µM, and had a more potent proliferation inhibitory effect than the positive control doxorubicine in 14 of 24 cell lines (Table 1). Since human hepatocellular cancer cell line was most sensitive to YLT322, we chose this cell line for further experiments. The viability of the human hepatocellular cancer cell lines exposed to increasing concentrations of YLT322 up to 5 µM for 24–72 hours was attenuated with increasing concentration and duration of exposure (Fig. 2A). These data directly suggest that YLT322 exerts a more potent growth inhibitory effect than doxorubicine and can inhibit hepatic carcinoma cell proliferation in a concentration- and time-dependent manner. In addition to these experiments, we have also examined the effects of YLT322 on cell viability using clonogenic assays as described below.

**Table 1:** The proliferation inhibition of YLT322 in human tumor cell lines.

Cancer type	Cell line	YLT322 (µM)	Doxorubicine (µM)
Liver	HepG2	0.39	1.50
Liver	Bel-7402	1.04	1.92
Liver	Bel-7404	0.65	3.02
Liver	SMMC-7721	3.34	9.46
Melanoma	A375	0.58	0.32
Melanoma	A2058	2.70	0.31
Colon	HCT116	1.35	5.50
Colon	SW480	2.46	3.38
Colon	SW620	0.65	0.35
Colon	LoVo	1.12	1.20
Colon	Colo205	2.90	0.60
Pancreas	PANC-1	1.36	1.70
Pancreas	BXPC-3	0.66	0.31
Lung	A549	1.65	8.60
Lung	SPC-A1	7.70	0.35
Lung	PC9	2.80	1.60
Lung	NCI-H1975	1.45	0.78
Ovary	SKOV3	1.87	1.10
Breast	MCF-7	1.90	5.80
Breast	MDA-MB-231	0.91	2.60
Breast	BT474	4.80	1.60
Breast	SK-RB-3	2.55	4.50
Cervix	HeLa	0.93	14.5
Epidermal	A431	2.70	4.20

Table 1. IC_50_ values (µM) for inhibition of cell proliferation by 48- hour treatment with YLT322 (0–20 µM) or doxorubicine (0–40 µ M). Data are expressed as the mean from three experiments.

### Inhibition of cell growth/survival by clonogenic assay

To further determine the effect of YLT322 on cell growth, we conducted clonogenic assay after YLT322 treatment. Fig. 2B and 2C present a significant inhibition of colony formation of HepG2 and Bel-7402 cell lines in a concentration-dependent manner after exposure to YLT322 compared with vehicle. When YLT322 concentration was at 1 µM, nearly no colony formation was detected. Overall, the results from clonogenic assay were consistent with the MTT data as shown in Fig. 2A, suggesting that YLT322 can inhibit cell growth in HepG2 and Bel-7402 hepatocellular cancer cell lines.

### Induction of apoptosis by YLT322

**Figure 3:**
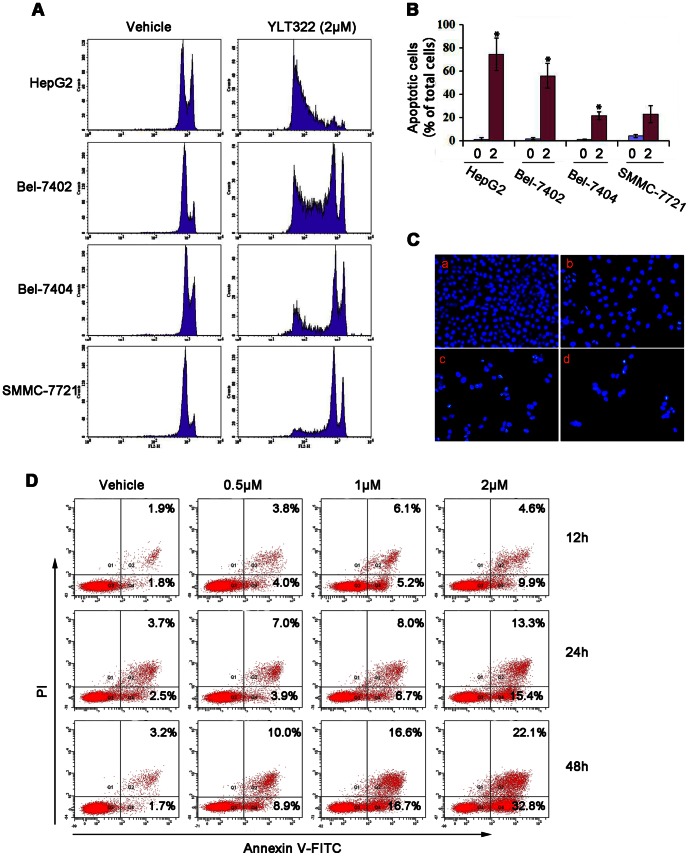
The effect of YLT322 on cell morphology and viability of cancer cells. **A.** Flow cytometric analysis of PI-stained HCC cell lines, including HepG2, SMMC-7721, Bel-7402 and Bel-7404, after treatment with 2 µM YLT322 for 48 hours. **B.** Statistical results of apoptosis assays presented as surviving cells (percentage of untreated control). Data are expressed as mean ± SD. for at least 3 independent experiments. (*p<0.05; **p<0.01; ***p<0.001). **C.** Fluorescence microscopy analysis of Hoechst 33342-stained HepG2 cells after incubation with varying concentrations of YLT322 (0 µM (a), 0.5 µM (b), 1 µM (c), 2 µM (d)) for 24 hours (×40). **D.** Flow cytometric analysis of cells stained with Annexin V-FITC/PI after treatment with various concentrations (0 µM, 0.5 µM, 1 µM, 2 µM) of YLT322 for 12 to 48 hours.

We next studied the induction of apoptosis in hepatocellular cancer cells treated with YLT322 to determine whether inhibition of cell viability is associated with activation of the programmed cell death pathways. As Fig. 3A and B indicates, YLT322 treatment increased the percentage of Sub-G1 cells from 3.2% in non-treated group to 88.5% in 2 µM YLT322-treated group in HepG2 cells. Similarly, in other hepatocellular cancer cell lines such as Bel-7402, Bel-7404 and SMMC-7721, the number increased from 1.8% to 55.3%, from 1.1% to 24.6%, and from 4.2% to 20.8%, respectively. Furthermore, the HepG2 cells exhibited features of apoptotic cells as revealed by Hoechst 33342 staining, including bright-blue fluorescent condensed nuclei, reduction of cell volume and nuclear fragmentation (Fig. 3C).

To confirm this cell death result, we also used other methods to detect apoptosis: Annexin V – FITC and PI fluorescence staining was analyzed by flow cytometry. Fig. 3D shows the time- and dose-dependent changes in the percentage of apoptotic cells when cells were exposed to 0.5 µM, 1 µM or 2 µM YLT322 for 12, 24 and 48 hours. After 48 hours, we found an obvious concentration-dependent reduction in the percentage of surviving cells (from 81.1% at 0.5 µM, to 66.7% at 1 µM, to 45.1% at 2 µM), with nearly a 40% change between the lowest and highest concentration. However, the earlier time points did not present significant changes, which was consistent with the MTT data as shown in Fig. 2A.

### Effect of YLT322 on the intrinsic apoptosis pathway

**Figure 4:**
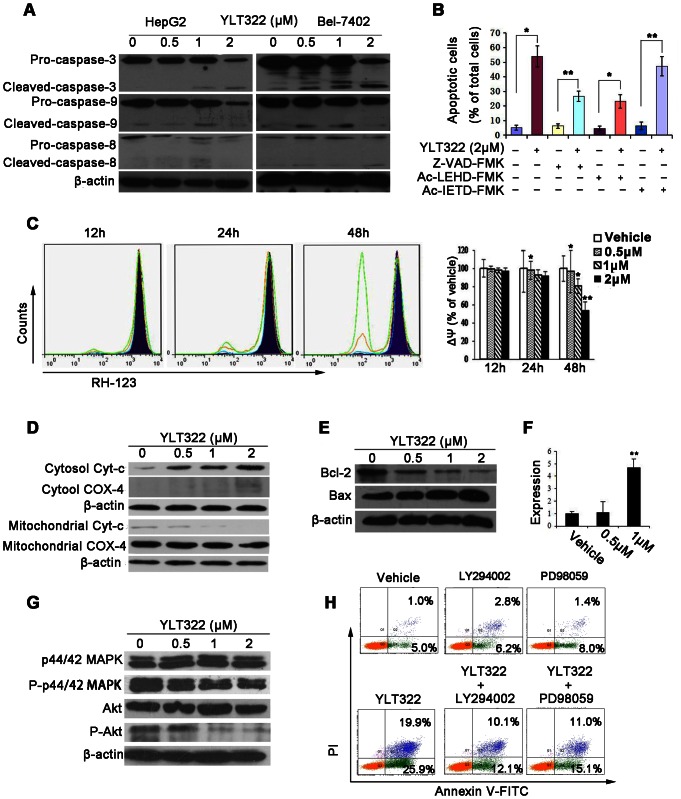
Effect of YLT322 on the expression levels of apoptosis-related proteins and the mitochondrial membrane potential (ΔΨ) of HepG2 cells. **A.** The expression levels of pro-caspase-8, -3, -9, and their activated forms in HepG2 and Bel-7402 cells treated with YLT322 for 48 hours as assayed by western blot. **B.** HepG2 cells were treated with 2 µM YLT322 alone or in combination with Z-VAD-FMK (a general caspase inhibitor), Ac-LEHD-FMK (caspase-9 inhibitor) or Ac-IETD-FMK (caspase-8 inhibitor). Data are expressed as mean ± SD. for at least 3 independent experiments. (*p<0.05; **p<0.01; ***p<0.001). **C.** The mitochondrial membrane potential (ΔΨ) was detected by RH123 staining after treatment with YLT322 at varying concentrations of 0 µM (Dark blue), 0.5 µM (blue), 1 µM (orange), 2 µM (green) for 12 to 48 hours (Bars, SD; Column, mean; n, 3;*p<0.05; **p<0.01; ***p<0.001). **D.** The level of cytochrome *c* in the cytosol and mitochondrial fraction was analyzed by western blot analysis after incubation of cells with YLT322 for 48 hours. Expression of COX-4 served as the loading control for the mitochondria fraction. **E.** The protein levels of Bcl-2 and Bax were detected by western blot in HepG2 cells treated with YLT322 for 48 h. **F.** The mRNA level of Bax was examined by real-time RT-PCR in HepG2 cells treated with YLT322 for 48 h. (Bars, SD; Column, mean; n, 3;*p<0.05; **p<0.01; ***p<0.001). **G.** The expression levels of Akt, p44/42 MAPK and their phosphorylated forms were assayed by western blot in HepG2 cells treated with YLT322. **H.** HepG2 cells were treated with 2 µM YLT322 alone or in combination with LY294002 (PI3K/AKT inhibitor) or PD98059 (MEK/ERK inhibitor). Data shown are representative of three independent experiments.

Apoptosis is associated with the activation of specific caspase cleavage cascades. To further characterize the apoptosis pathways stimulated by YLT322, we analyzed the proteolytic processing of caspase-3, caspase-8 and caspase-9. A reduction in pro-caspases-9 and -3 and an increase in the levels of their cleaved forms were observed following exposure to YLT322 for 48 hours in HepG2 and Bel-7402 cells. The effect on the level of pro-caspase-8 protein was also observed, but the increase in cleaved-caspase-8 was not significant (Fig. 4A). To explore whether YLT322-induced apoptosis is specifically associated with caspase activation and to determine which type of apoptotic pathways is predominant, we examined by FCM whether Z-VAD-FMK (a general caspase inhibitor), Ac-LEHD-FMK (caspase-9 inhibitor) and Ac-IETD-FMK (caspase-8 inhibitor) can affect the extent of YLT322-induced apoptosis. As shown in Fig. 4B, compared with YLT322 treatment alone, treatment with 2 µM YLT322 combined with 20 µM Z-VAD-FMK decreased the percentage of apoptotic cells from 46.2% to 26.9% while that with 50 µM Ac-LEHD-FMK decreased the percentage of apoptotic cells to 22.6%, . However, the combination of YLT322 with 50 µM Ac-IETD-FMK had only a slight effect on YLT322-induced apoptosis. These results indicate that the caspase family of proteins plays an important role in YLT322-induced apoptosis, and that the intrinsic pathway contributes more to YLT322-induced apoptosis than the extrinsic pathway.

### Effects of YLT322 on cytochrome c and ΔΨ

A limiting step in the intrinsic apoptotic pathway is the damage of mitochondria and the release of cytochrome *c* from mitochondria into the cytosol. To observe the change in ΔΨ in cells exposed to YLT322, a mitochondria-specific and voltage-dependent dye RH123 was used. A time- and dose-dependent reduction in ΔΨ was observed when cells (HepG2) were exposed to 0.5 µM, 1 µM, or 2 µM YLT322 for 12, 24, and 48 hours (Fig. 4C). After 48 hours, there was a significant dose-dependent reduction in the ΔΨ of more than 40.0% in the range of 0.5 to 2 µM. However, at 12 and 24 h, the loss of ΔΨ was indiscernible, which was in line with the Annexin V – FITC/PI data. Protein analysis showed that after YLT322 treatment for 48 h, cytochrome c level was increased in the cytosolic fractions but decreased in the mitochondria fraction (Fig. 4D).

### Effect of YLT322 on the expression levels of Bax and Bcl-2

Bcl-2 family proteins are essential for regulating mitochondrial integrity through the balance between anti-apoptotic and pro-apoptotic members [16]. We examined by western blot analysis the expression of some Bcl-2 family proteins in HepG2 cells after YLT322 treatment for 48 hours. As shown in Fig. 4E, the expression of Bcl-2 significantly decreased in a concentration-dependent manner while that of Bax was increased. Furthermore, real-time reverse transcription- PCR (Fig. 4F) revealed that the Bax mRNA was up-regulated by YLT322, which was consistent with the result of western blot analysis in Fig. 4E.

### Effect of YLT322 on p44/42 MAPK and Akt signaling pathways

We next investigated whether p44/42 MAPK and Akt, which are considered to be important for cell proliferation and apoptosis, are involved in YLT322-mediated apoptosis [17]. We found that YLT322 decreased the expression of phosphorylated Akt and phosphorylated-MAPK without affecting their total expression level (Fig. 4G). Next, cells pretreated with the PI3K/AKT inhibitor LY294002 and MEK/ERK inhibitor PD98059 were exposed to 2 µM YLT322 for 48 hours. As shown in Fig. 4H, LY294002 and PD98059 reduced YLT322-induced cell apoptosis from 45.8% to 22.2% and to 26.1%, respectively. This suggests that p44/42 MAPK and Akt play an important role in YLT322-induced apoptosis.

### Anti-tumor efficacy of YLT322 in human tumor xenograft models

**Figure 5:**
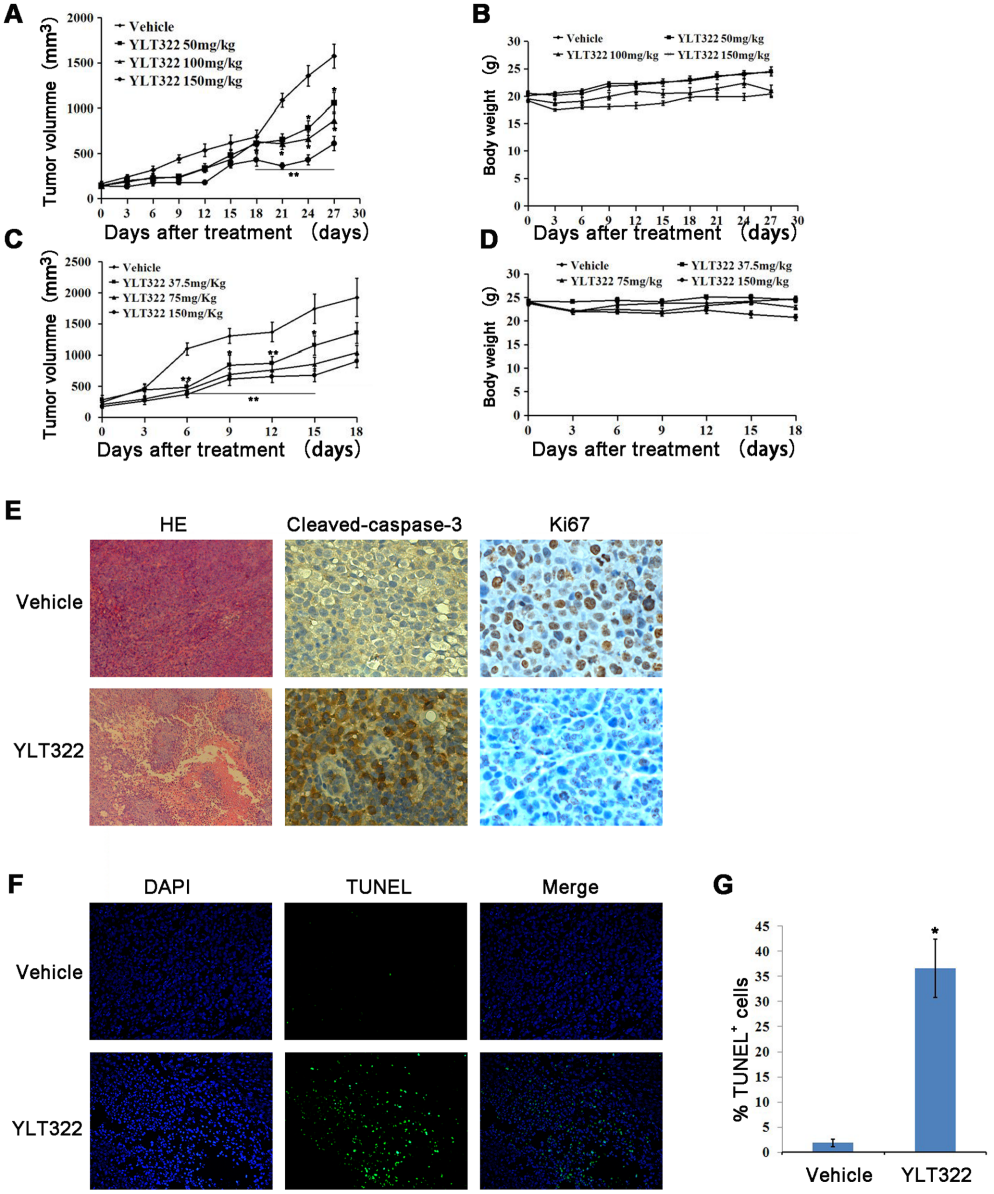
The effect of YLT322 on HepG2 and HCT116 tumor xenografts *in vivo*. Animals were given YLT322 or vehicle by intraperitoneal injection once daily (qd) at the indicated dosage levels. The tumor volume and animal weight of the HepG2 (**A** and **B**) and HCT116 (**C** and **D**) xenograft models were measured twice weekly. Points, mean tumor volume (mm^3^); bars, SE; n, 6; *p<0.05; **p<0.01; ***p<0.001. (**E** and **F**) After 6 days of YLT322 treatment, the HepG2 tumors treated with 150 mg/kg YLT322 or vehicle were collected separately (three per group). The sections containing tumors were examined by immunohistochemical analysis with anti-Ki67 (×40) and caspase-3 antibody (×40), and by hematoxylin staining (×5). TUNEL detection was performed for apoptosis analysis (×20). **G**. The percentage of apoptosis in each group. TUNEL-positive cells were counted in five high power fields/slide, and data were summarized in terms of percent positive cells. Bars, SEM; columns, mean; *p<0.05; **p<0.01; ***p<0.001.

To assess the antitumor effect of YLT322 *in vivo*, HepG2 and HCT116 cells were injected subcutaneously into the right flanks of BALB/c nude mice to establish xenografts. Tumor-bearing mice were administered with vehicle or YLT322 at a dosage of 50, 100 or 150 mg/kg/day for the HepG2 model and 37.5, 75 or 150 mg/kg/day for the HCT116 model. YLT322 substantially suppressed tumor growth in a dose-dependent manner, with the inhibition of tumor progression at 66.7% in the HepG2 model treated with 150 mg/kg YLT322 and at 56.7% in the HCT116 model treated with 150 mg/kg YLT322 (Fig. 5A,C). Furthermore, YLT322 treatment was well tolerated and did not cause significant loss in body weight (Fig. 5B,D).

To further demonstrate that tumor growth inhibition *in vivo* is a result of apoptosis, histological and immunohistochemical analyses were performed on tumor tissues isolated from HepG2 tumor model. As shown in Fig. 5E, 5F, hematoxylin and eosin staining revealed a significant regression of tumors isolated from YLT322-treated animals which was not observed in the vehicle group. Meanwhile, YLT322 treatment caused a significant decrease in the number of Ki67-positive cells, but an increase in the number of caspase-3-positive cells. These changes were not observed in the vehicle group. The percentage of TUNEL-positive cells in YLT322-treated tumors was higher (36.6%) than that in the vehicle group(7.6%)(Fig. 5G). Taken together, these results clearly show that YLT322 inhibits tumor growth *in vivo* through a reduction in proliferating cells and increased apoptosis in human tumor xenograft model.

### Toxicity Evaluation

**Figure 6:**
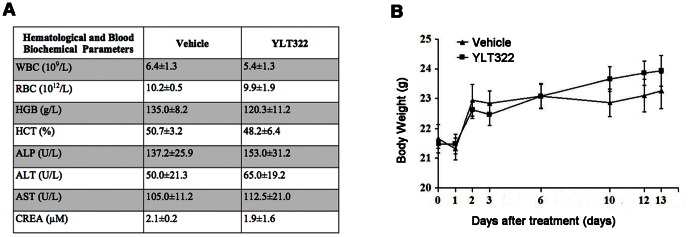
Evaluation of side effects of YLT322 in mice. **A.** Hematological and serum biochemical values of mice at day 13 (n = 5 for both control and treated). **B.** Data showing average body weight changes in the control (n = 5) and YLT322 treated mice (n = 5). Values are indicated in mean
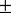
SEM.

For a preliminary safety estimate of YLT322, a sub-acute toxicity test was performed. No significant change was observed in hematological and serum biochemical values (Fig. 6A) and body weight (Fig. 6B) in YLT322-treated mice compared to control.

## Discussion

Dysregulation of the programmed cell death process (apoptosis) is a hallmark of many types of malignancies and the cause of cancer cell resistance to chemotherapy [7], [18], [19]. Thus restoring apoptosis is a promising strategy for effectively treating cancers [20]. Currently, many therapeutic approaches, including various biologicals, hormones, and small molecule chemotherapeutic agents such as ABT737, AT101, HS-113 and chloroquine, inhibit tumors by triggering cell apoptosis [18], [21]–[24]. Previous studies have demonstrated that some benzothiazole derivatives can induce apoptosis [10], [13], but the mechanism has not been clearly determined. In the present study, our results show that YLT322 is a broad spectrum anti-cancer compound with high apoptotic-inducing activity to a panel of cancer cells with the most potent effect observed with HepG2. Moreover, the anti-cancer effect of YLT322 was demonstrated to be associated with the induction of apoptosis.

Possible drug targets for regulating cell apoptosis have been widely studied [7]. In general, the action of apoptosis is through two very distinct signaling pathways: intrinsic pathway or extrinsic pathway [25], [26]. Caspase-3, the major effector caspase, is responsible for the cleavage of the majority of polypeptides that undergo proteolysis in an apoptotic cell and plays a central role in both types of apoptotic pathways [27]. It is activated by upstream effector proteins including caspase-8 and caspase-9, the apical proteases in the extrinsic and intrinsic pathways, respectively [7], [28]. After YLT322 treatment, we observed in cells a reduction in procaspase-3, -8 and -9 and a concomitant increase in their cleavage, indicative of enzyme activation,. Addition of the irreversible inhibitors, Z-VAD-FMK (caspase inhibitors) and Ac-LETD-FMK(caspase-9 inhibitors) significantly decreased the percentage of apoptotic cells after treatment with YLT322, suggesting that cell apoptosis induced by YLT322 may be dependent on caspase activation. However, Ac-IETD-FMK, a caspase-8 inhibitor, was unable to antagonize YLT322-induced cell death. It is possible that caspase-8 plays a non-apoptotic role in the cell, such as regulating cell differentiation and senescence [29]. We assumed that the activation of caspase-8 observed after YLT322 treatment is a possible downstream “bystander” event which has been reported elsewhere [30]. These data suggest that the intrinsic apoptosis pathway is more important than the extrinsic pathway in apoptosis induced by YLT322.

A key step in the intrinsic apoptotic pathway is the disruption of the mitochondrial membrane, which triggers the release of cytochrome *c* from mitochondria to cytosol. From our study, a reduction in ΔΨ and a release of cytochrome *c* from the mitochondria into the cytosol were observed in a dose-dependent manner after YLT322 treatment.

Bcl-2 protein family plays an important role in the regulation of mitochondrial apoptosis pathway [7]. Multidomain pro-apoptotic proteins such as Bax and Bid can open the ion channel in the outer mitochondrial membrane in the presence of a death signal, leading to mitochondrial membrane permeabilization and release of cytochrome *c* into the cytosol [31], [32]. Cytochrome *c* in the cytosol binds to the scaffolding protein Apaf-1 and pro-caspase-9 to form the apoptosome, which triggers the apoptotic cascade reaction thereafter. The anti-apoptotic Bcl-2 family members such as Bcl-2 and Bcl-xl inhibit cytochrome *c* release by blocking the activation of pro-apoptotic proteins. For most cancers, the over-expression of Bcl-2 protein associates with poor survival, uncontrolled progression and resistance to anticancer agents, making Bcl-2 family members promising anticancer drug targets [24], [33]–[34]. Our results showed that the expression of Bcl-2 decreased in cells treated with YLT322, whereas that of Bax increased. We assumed that cytochrome *c* release from the mitochondria into the cytosol upon YLT322 treatment is due to the down-regulation of Bcl-2 and/or up-regulation of Bax.

Studies have suggested that Akt/p44/42 MAPK pathway plays essential roles in apoptosis and could be modulated for cancer prevention and treatment [35]–[37]. Here, we found that treatment with YLT322 decreased the phosphorylation of both Akt and p44/42 MAPK. This suggests that Akt and p44/42 MAPK may contribute to the apoptosis induced by YLT322.

YLT322 treatment on nude mice bearing tumors resulted in significant inhibition of tumor volume without any apparent sign of toxicity. Immunohistochemical studies revealed that the regression of tumor size in mice models by YLT322 was also associated with the activation of apoptosis, as demonstrated by of the presence of active caspase-3-positive and TUNEL-positive cells in tumor xenograft samples. YLT322 also caused a decrease in Ki67-positive cells, suggesting a reduction in cell proliferation.

In conclusion, our study demonstrated that YLT322 inhibits cell growth/proliferation by inducing apoptosis *via* the intrinsic pathway. In addition, YLT322 suppressed the growth of human liver cancer xenografts without significant toxicity, suggesting that it may be a potential candidate for cancer therapy. In depth studies are still needed to elucidate the precise targets mediating the anti-cancer effects of YLT322.
